# Effect of atorvastatin on cardiomyocyte hypertrophy through suppressing MURC induced by volume overload and cyclic stretch

**DOI:** 10.1111/jcmm.14044

**Published:** 2018-12-03

**Authors:** Wen‐Pin Cheng, Huey‐Ming Lo, Bao‐Wei Wang, Su‐Kiat Chua, Kou‐Gi Shyu

**Affiliations:** ^1^ Department of Medical Education and Research Shin Kong Wu Ho‐Su Memorial Hospital Taipei Taiwan; ^2^ Division of Cardiology Shin Kong Wu Ho‐Su Memorial Hospital Taipei Taiwan; ^3^ School of Medicine Fu‐Jen Catholic University New Taipei City Taiwan; ^4^ Department of General Medicine Shin Kong Wu Ho‐Su Memorial Hospital Taipei Taiwan

**Keywords:** atorvastatin, cyclic stretch, MURC, volume overload

## Abstract

MURC (muscle‐restricted coiled‐coil protein) is a hypertrophy‐related gene. Hypertrophy can be induced by mechanical stress. The purpose of this research was to investigate the hypothesis that MURC mediates hypertrophy in cardiomyocytes under mechanical stress. We used the in vivo model of an aortocaval shunt (AV shunt) in adult Wistar rats to induce myocardial hypertrophy. We also used the in vitro model of cyclic stretch in rat neonatal cardiomyocytes to clarify MURC expression and the molecular regulation mechanism. The flexible membrane culture plate seeding with cardiomyocytes Cardiomyocytes seeded on a flexible membrane culture plate were stretched by vacuum pressure to 20% of maximum elongation at 60 cycles/min. AV shunt induction enhanced MURC protein expression in the left ventricular myocardium. Treatment with atorvastatin inhibited the hypertrophy induced by the AV shunt. Cyclic stretch markedly enhanced MURC protein and mRNA expression in cardiomyocytes. Addition of extracellular‐signal‐regulated kinase (ERK) inhibitor PD98059, ERK small interfering RNA (siRNA), angiotensin II (Ang II) antibody and atorvastatin before stretch, abolished the induction of MURC protein. An electrophoretic mobility shift assay showed that stretch enhanced the DNA binding activity of serum response factor. Stretch increased but MURC mutant plasmid, ERK siRNA, Ang II antibody and atorvastatin reversed the transcriptional activity of MURC induced by stretch. Adding Ang II to the cardiomyocytes also induced MURC protein expression. MURC siRNA and atorvastatin inhibited the hypertrophic marker and protein synthesis induced by stretch. Treatment with atorvastatin reversed MURC expression and hypertrophy under volume overload and cyclic stretch.

## 1 INTRODUCTION

Cardiac hypertrophy is an adaptive mechanism of increase in cardiomyocyte size under haemodynamic overload of the heart.^1^ Haemodynamic load is a crucial regulator of cardiac function and gene expression. If the haemodynamic overload is prolonged, hypertrophy ultimately leads to heart failure and death. There are two types of hypertrophy in response to haemodynamic overload. Pressure overload‐mediated concentric hypertrophy leads to a normal left ventricular volume and an increase in wall thickness.^2^ However, the eccentric hypertrophy induced by volume overload causes an increase in the left ventricular volume while not affecting the wall thickness.^3^ Recently, there has been a significant increase in the number of studies concerned with the molecular mechanism of hypertrophy, especially in concentric hypertrophy induced by pressure overload.^4^ However, the molecular regulation mechanism of eccentric hypertrophy caused by volume overload is still not fully understood.^5^

MURC (a muscle‐restricted coiled‐coil protein), also called Cavin‐4 (caveolae‐associated protein 4), is a cytosolic protein and the fourth member of the Cavin family.^6^ A recent study showed that MURC could interact with Cavin‐1, Cavin‐2 and Cavin‐3 to form caveolae. Overexpression of MURC swelled the caveolae but was not crucial to caveolae formation.^7^ MURC was found to be expressed in vascular smooth muscle cells, skeletal muscle cells and cardiomyocytes.^8^ A study suggested that pressure overload could induce MURC messenger RNA (mRNA) expression in cardiomyocytes.^9^ Overexpression of MURC in a transgenic mouse model led to atrial fibrillation and chronic heart failure.^10^ Ogata et  al reported that MURC was involved in the cardiac concentric hypertrophy under pressure overload.^11^

No conclusive proof has been obtained of how volume overload caused by aortocaval (AV) shunt affects MURC upon hypertrophy in the myocardium. This study was designed to clarify whether an AV shunt can regulate myocardial MURC expression and the molecular regulation mechanisms mediating MURC expression under stretch in cardiomyocytes. Atorvastatin was previously indicated to protect against hypertrophy. We thus also investigated the role of atorvastatin in the treatment of cardiac myocyte hypertrophy under AV shunt and cyclic stretch conditions.

## 2 MATERIALS AND METHODS

### 2.1 Ethics statement

Male Wistar rats purchased from BioLASCO (Yilan, Taiwan) were fed and housed with toys, auditory, visual and hideaway enrichment in accordance with the standards of the Committee of Animal Care and Use of Shin KongWu Ho‐Su Memorial Hospital. All animal study protocols were approved by this committee (permit No. 1021025015) and carried out in accordance with the National Institutes of Health (NIH) Guide for the Care and Use of Laboratory Animals (NIH Publication No. 86‐23, revised 2011). The animal study was performed after a fully anaesthetized state was confirmed (ie, no response to toe pinching). All efforts were made with consideration of the animal's welfare and to minimize their suffering according to the guidelines of our institution's institutional animal care and use committee.

### 2.2 Aortocaval shunt model

On the day of surgery, the male Wistar rats (weight range from 250 to 300 g) were anaesthetized with isoflurane (80 mg/kg). The vena cava and aorta were exposed through an abdominal midline incision after a fully anaesthetized state (ie, no response to toe pinching). In brief, with an 18‐gauge disposable needle connected to a plastic syringe, the aorta was punctured at the union of the segment two‐thirds caudal to the left renal artery and one‐third cephalic to the aortic bifurcation. Sham‐operated control animals were prepared in a similar manner, except that the aorta was not punctured. For the AV shunt time‐course study, rats were randomly divided into five groups: (a) Sham‐operated (n = 7), (b) AV shunt 3 days (n = 7), (c) AV shunt 7 days (n = 7), (d) AV shunt 10 days (n = 7), (e) AV shunt 14 days (n = 7). For the AV shunt and atorvastatin treatment study, rats were randomly divided into four groups: (a) Sham‐operated (n = 7), (b) Sham‐operated and atorvastatin treatment (n = 7), (c) AV shunt 10 days (n = 7), (d) AV shunt 10 days and atorvastatin treatment (n = 7). Atorvastatin at 30 mg/kg/d was given for 10 days after induction of an AV shunt. At the end of experiment, rats were killed with an overdose of isoflurane. Left ventricular tissue was obtained for Western blot analysis. We monitored the condition of rats twice a day after surgery.

### 2.3 Cardiomyocyte primary culture

Cardiomyocytes were obtained from 2–3‐day‐old Wistar rats through trypsinization as previously described.^12^ Cultured cardiomyocytes were more than 95% pure, as revealed by observing their contractile characteristics using a light microscope and staining with an anti‐desmin antibody (Dako Cytomation, Glostup, Denmark). Cardiomyocytes were seeded on a flexible membranes base of six culture wells in Ham's F‐10 medium containing 20% foetal bovine serum. After 3 days in culture, cells were transferred to serum‐free Dulbecco's modified Eagle's medium and subjected to stretch.

### 2.4 Reverse transcription polymerase chain reaction

Reverse transcription polymerase chain reaction (PCR) was performed as previously described.^12^

### 2.5 Real‐time quantitative polymerase chain reaction

Real‐time quantitative PCR was performed as previously described.^12^ The primers used were as follows: MURC, 5’‐d(CCGCATCCCTGTCTGTTGTT)‐3’ (forward) and 5’‐d(TTCCTCAGCTTCCTC CTCGTT)‐3’(reverse) and *α*‐tubulin, 5’‐d(ATCACCAATGCTTGCTTTGAG)‐3’ (forward) and 5’‐d(CAGCATCTTCCTTGCCTGTGA)‐3’ (reverse).

### 2.6 Western blot analysis

We used Western blotting to detect protein levels as previously described.^12^ The antibodies used for the Western blot analysis were anti‐MURC (1:1000 dilution; Sigma‐Aldrich, St. Louis, MO, USA), anti‐β‐MHC (β‐myosin heavy chain) and anti‐BNP (B‐type natriuretic peptide) (1:200 dilution; both from Santa Cruz Biotechnology, Dallas, TX, USA).

### 2.7 Immunohistochemistry

Immunohistochemistry was performed as previously described.^13^ The antibodies used for immunohistochemistry were rabbit anti‐MURC (Santa Cruz Biotechnology), rabbit anti‐β‐MHC (Santa Cruz Biotechnology) and mouse anti‐desmin (Santa Cruz Biotechnology) at 1:500 overnight at 4°C, followed by incubation with donkey anti‐rabbit (fluorescein isothiocyanate) immunoglobulin G (IgG) and donkey anti‐mouse (tetramethylrhodamine) IgG (Jackson Immuno Research Laboratories, West Grove, PA, USA) at 1:500 for 60 minutes. Fluorescent signals were captured using a confocal microscope (Nikon Digital Eclipse; Nikon Instruments, Melville, NY, USA) and assayed using the microscope associated image processing and analysis software.

### 2.8 In vitro cyclic stretch on cultured cardiomyocytes

The Flexcell FX‐2000 strain unit, (Flexcell International, Burlington, NC, USA) has been characterized and described in detail elsewhere.^12^ To investigate the roles of ERK (extracellular signal‐regulated kinase), p38 and JNK (c‐Jun N‐terminal kinase) MAPK (mitogen‐activated protein kinase) in the protein expression of MURC induced by stretch, cardiomyocytes were treated with SP600125 (20 μmol L^−1^; Calbiochem, San Diego, CA, USA), SB203580 (3 μmol L^−1^; Calbiochem) or PD98059 (50 μmol L^−1^; Calbiochem) 30 minutes before stretch.

### 2.9 Electrophoretic mobility shift assay

An electrophoretic mobility shift assay was used to detect the DNA‐protein binding activity as previously described.^13^ The serum response factor (SRF) binding site used was CCATATTAGG. The mutant oligonucleotide of SRF sequences were TCATAATATT.^13^

### 2.10 Construction of small interfering RNA

Cardiomyocytes were transfected with 800 ng of small interfering RNA (siRNA) (Dharmacon, Lafayette, CO, USA) to knock down gene expression. The MURC siRNA sequence used was as follows: sense, 5’‐GCAACACGGGCUACGUUGU‐3’ and antisense, 5’‐ACAACGUAGCCCGUGUUGC‐3’. For a negative control, green fluorescent protein double‐stranded RNA interference was used, sense, 5’‐PGGCUACGUCCAGGAGCGCACC‐3’ and antisense, 5’‐PUGCGCUCCUGGACGUAGCCUU‐3’ (Dharmacon). For ERK siRNAs, the sequence used was as follows: sense, 5’‐GACCGGAUGUUAACCUUUAUU‐3’ and antisense, 5’‐PUAAAGGUUAACAUCCGGUCUU‐3’. (Dharmacon).

### 2.11 Promoter activity assay

We used T‐Pro NTR II transfection reagent (T‐Pro Biotechnology, Taipei, Taiwan) to transform the MURC promoter construct containing the SRF binding site into cardiomyocytes. MURC promoter activity was measured as previously described.^13^

### 2.12 Determination of angiotensin II by enzyme‐linked immunosorbent assay

We collected the conditioned medium from cardiomyocytes subjected to stretch and from control (non‐stretched) cells for detection of angiotensin (Ang II).

### 2.13 Detection of protein synthesis

We detected [^3^H]proline incorporation into the cells to determine protein synthesis as previously described.^13^

### 2.14 Statistical analysis

All results are expressed as the mean ± standard error of the mean. Statistical significance was evaluated using analysis of variance (ANOVA) (GraphPad Software, La Jolla, CA, USA). Dunnett's test was used to compare multiple groups with a single control group. The Tukey‐Kramer comparison was used for pairwise comparisons between multiple groups after ANOVA *P < *0.05 was considered to denote statistical significance.

## 3 RESULTS

### 3.1 AV shunt increased myocardial MURC protein and mRNA expression

**Figure 1:**
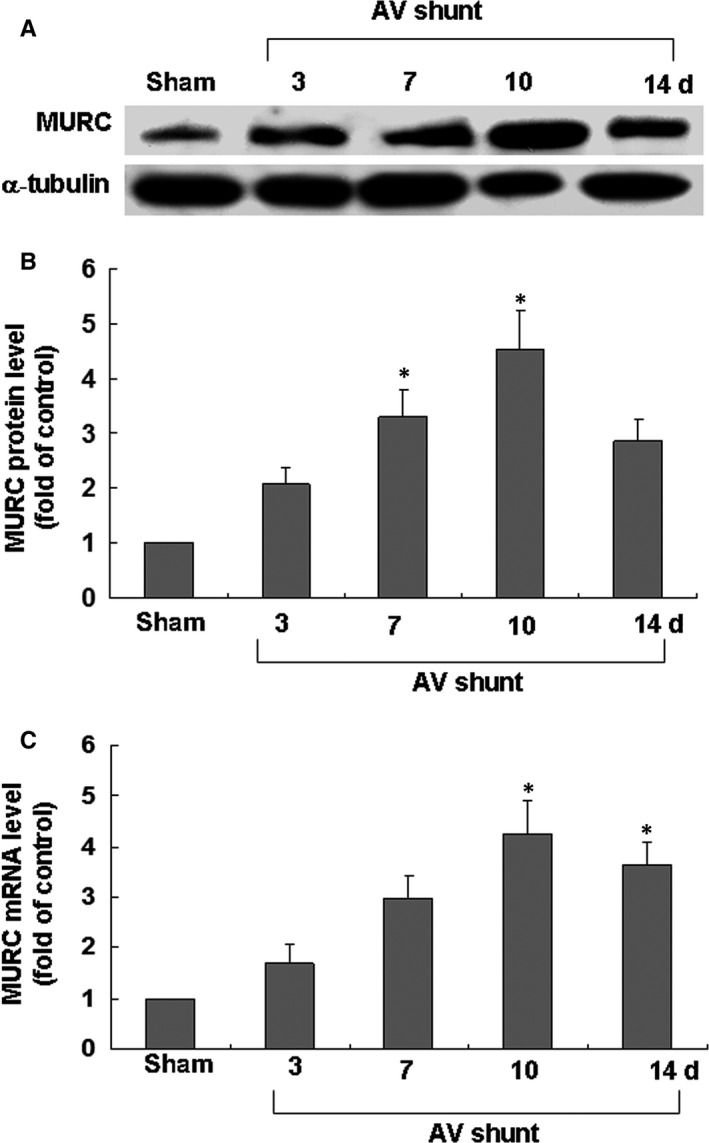
Effect of aortocaval shunt (AV shunt) on myocardial MURC protein levels and mRNA. A, Representative Western blots for MURC in rat myocardium after AV shunt. B, Quantitative analysis of MURC protein levels. The values of MURC protein after AV shunt were normalized to match the α‐tubulin measurement and then expressed as a ratio of normalized values to protein in the sham group (n = 3 per group). *P < 0.05 vs sham group. C, Fold increase in MURC mRNA as a result of induction of an AV shunt. The values for the experimental groups were normalized to match the GADPH measurement and then expressed as a ratio of normalized values to mRNA in the sham group. *P < 0.05 vs sham group (n = 3 per group).

We used the AV shunt to clarify whether myocardial MURC expression was elevated after volume‐overload. As shown in Figure 1A,B, myocardial MURC protein expression was significantly increased in rats under the AV shunt for 10 days. Real‐time PCR indicated that MURC mRNA was up‐regulated after induction of the AV shunt (Figure 1C). These results demonstrated that the AV shunt induced the myocardial MURC expression.

### 3.2 Atorvastatin inhibited myocardial MURC protein expression and hypertrophy induced by the AV shunt

**Figure 2:**
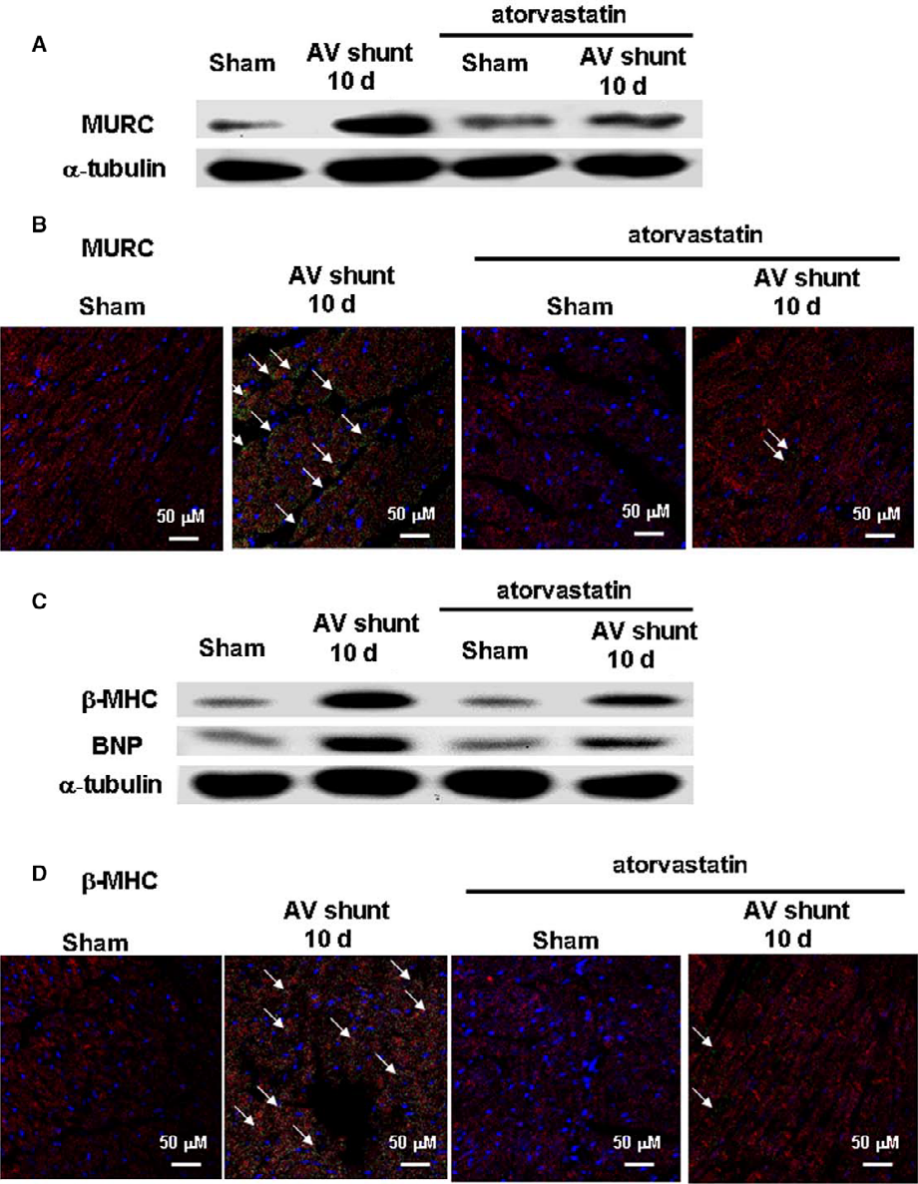
Effect of AV shunt and treatment with atorvastatin (30 mg/kg/d) on myocardial MURC protein levels and hypertrophy. A, Representative Western blots for MURC after induction of an AV shunt with or without treatment with atorvastatin. B, Representative microscopic images of cardiomyocytes 10 days after AV shunt induction. Addition of atorvastatin before AV shunt induction followed by staining with the anti‐MURC and anti‐desmin antibodies. Similar results were observed in two other independent experiments. Green colour and arrows indicate MURC positive cells. Blue colour indicates nucleus stained by Hoechst stain. Red colour indicates cytoskeleton stained by desmin. C, Representative Western blots with staining of β–MHC (β‐myosin heavy chain) and BNP (B‐type natriuretic peptide) antibodies after induction of an AV shunt with or without atorvastatin treatment. D, Representative microscopic images of cardiomyocytes 10 days after AV shunt induction. Addition of atorvastatin before AV shunt induction followed by staining with the anti‐β–MHC and anti‐desmin antibodies. Similar results were observed in another two independent experiments. Green colour and arrows indicate β–MHC positive cells. Blue colour indicates nucleus stained by Hoechst stain. Red colour indicates cytoskeleton stained by desmin.

Treatment with atorvastatin markedly reduced the elevation of MURC protein under the AV shunt (Figure 2A, Figure S1A and Figure 2B, Figure S1B). As illustrated in Figure 2C, Figure S1C and Figure 2D, Figure S1D, atorvastatin significantly inhibited the β‐MHC (a hypertrophic cardiomyocyte marker) induced by the AV shunt. Moreover, treatment with atorvastatin also significantly inhibited another hypertrophic marker, BNP protein expression, induced by the AV shunt (Figure 2C and Figure S1C). These results revealed that atorvastatin inhibited MURC protein expression and hypertrophy caused by the AV shunt in the myocardium.

### 3.3 Cyclic stretch enhanced the cardiomyocyte MURC protein and mRNA expression

**Figure 3:**
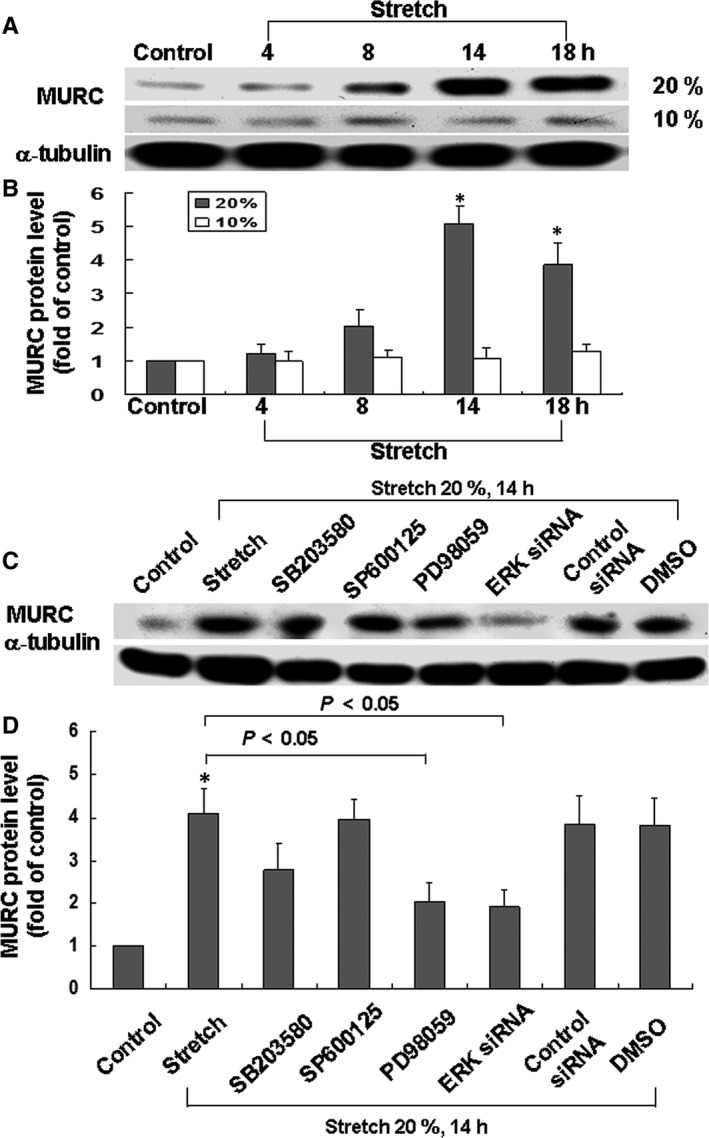
Effects of in vitro stretch and treatment with MAPK inhibitors on MURC protein expression in cardiomyocytes. A, Representative Western blots for MURC in cardiomyocytes subjected to cyclic stretch by 10% or 20% for various periods of time. B, Quantitative analysis of MURC protein levels. Values of MURC protein under stretch were normalized to match the α‐tubulin measurement and then expressed as a ratio of normalized values to protein in the control group (n = 3 per group). *P < 0.05 vs control. C, Representative Western blots for MURC protein levels in cardiomyocytes subjected to stretch in the absence or presence of MAPK inhibitors, small interfering RNA and vehicle (dimethyl sulfoxide 0.1%). D, Quantitative analysis of MURC protein levels. Values of MURC protein under stretch were normalized to match the *α*‐tubulin measurement and then expressed as a ratio of normalized values to protein in the control group (n = 3 per group). *P < 0.05 vs control.

As shown in Figure 3A,B, cyclic stretch significantly increased the level of MURC protein expression. The MURC protein expression under 10% elongation was similar to that of the control without stretch. The result of real‐time PCR indicated that MURC mRNA increased markedly after 8 hours of stretch at 20% (Figure S2). We demonstrated that cyclic stretch induced cardiomyocyte MURC expression.

### 3.4 Cardiomyocyte MURC protein expression induced by stretch was mediated by ERK

As shown in Figure 3C, the addition of PD98059 (50 μmol L^−1^) 30 minutes before stretch markedly inhibited the protein expression of MURC induced by cyclic stretch. However, treatment with SP600125 (20 μmol L^−1^) or SB203580 (3 μmol L^−1^) did not affect MURC protein expression. In addition, when we tested the specific effect of suppressing the ERK MAPK pathway on MURC expression, treatment with the ERK siRNA before stretch significantly blocked the MURC protein expression induced by stretch. Scrambled siRNA and dimethyl sulfoxide alone as a vehicle control had no effect on the MURC expression induced by cyclic stretch. Treatment with atorvastatin (10 μmol L^−1^) markedly inhibited the MURC protein expression induced by stretch (Figure S3). These findings indicated that the cyclic stretch induced cardiomyocyte MURC proteins through the ERK pathway.

### 3.5 Stretch enhanced SRF binding activity and MURC promoter activity

**Figure 4:**
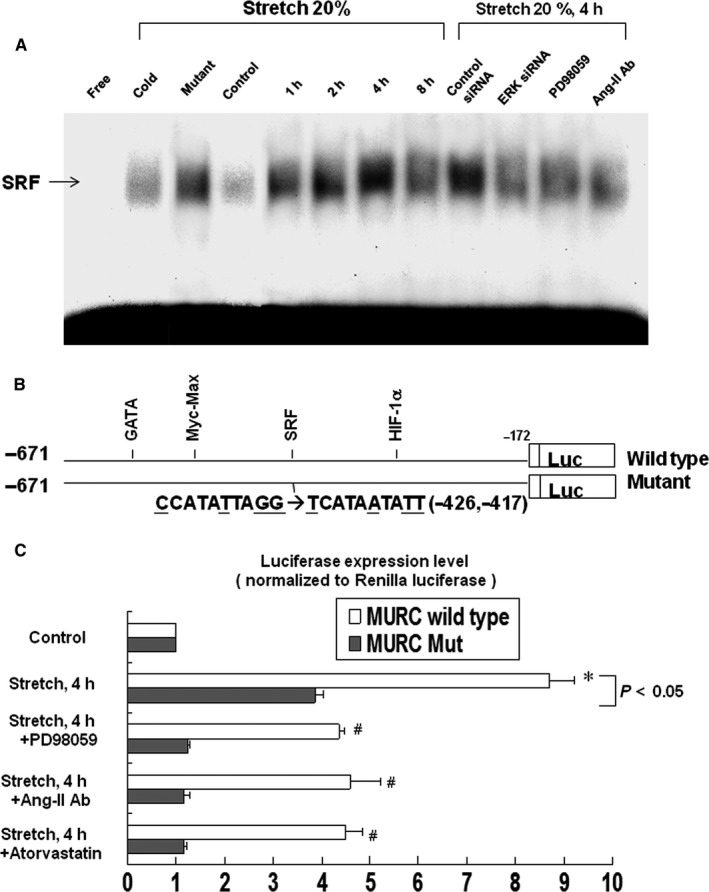
Effects of stretch on the serum response factor (SRF)‐binding activity and MURC promoter activity in cardiomyocytes. A, Representative electrophoretic mobility shift assay showing protein binding to SRF oligonucleotide in nuclear extracts of cardiomyocytes after stretch treatment for various times and in the absence or presence of extracellular signal‐regulated kinase (ERK) inhibitor or small interfering RNA (siRNA) and Ang II antibody. Arrows indicate the mobility of the complex. Similar results were found in another independent experiment. “Cold oligo” means unlabeled SRF oligonucleotide. B, Constructs of MURC promoter gene. C, Quantitative analysis of MURC promoter activity. Cardiomyocytes were transiently transfected with pMURC‐Luc using T‐Pro NTR II transfection reagent. The luciferase activity in cell lysates was measured and normalized with *Renilla *activity (n = 3 per group). *P < 0.05 vs control. ^#^ *P < *0.05 vs stretch 4 h

Stretch for 4 h markedly enhanced the DNA‐protein binding activity of SRF in cardiomyocytes (Figure 4A). An excess of unlabelled SRF oligonucleotide competed with the probe for binding SRF protein. However, an oligonucleotide containing a 4‐bp substitution at the SRF binding site did not compete for binding. Treatment with PD98059 and Ang II antibody (5 µg/mL, purchased from R&D Systems, Minneapolis, MN, USA) 30 minutes or ERK siRNA 24 hours before stretch significantly inhibited the DNA‐protein binding activity induced by stretch. We believe this implies that stretch increases SRF binding activity through Ang II and ERK in cardiomyocytes.

To investigate whether the MURC expression induced by cyclic stretch was regulated at the transcriptional level, we used a luciferase reporter assay to determine the genetic transcription activity of MURC in cardiomyocytes under stretch conditions. The MURC promoter construct contained SRF, GATA, MycMax and HIF‐1α (hypoxiainducible factor‐1α) binding sites (Figure 4B). As shown in Figure 4C, cyclic stretch markedly increased the promoter activity of MURC, but the MURC mutant did not have the same effect. Moreover, transient transfection of MURC‐mutant plasmid and treatment with PD98059, Ang II antibody or atorvastatin inhibited the promoter activity induced by stretch. Our results demonstrated that stretch‐induced MURC expression in cardiomyocytes occurred at the transcriptional level.

### 3.6 Stretch enhanced cardiomyocyte MURC protein expression through Ang II

**Figure 5:**
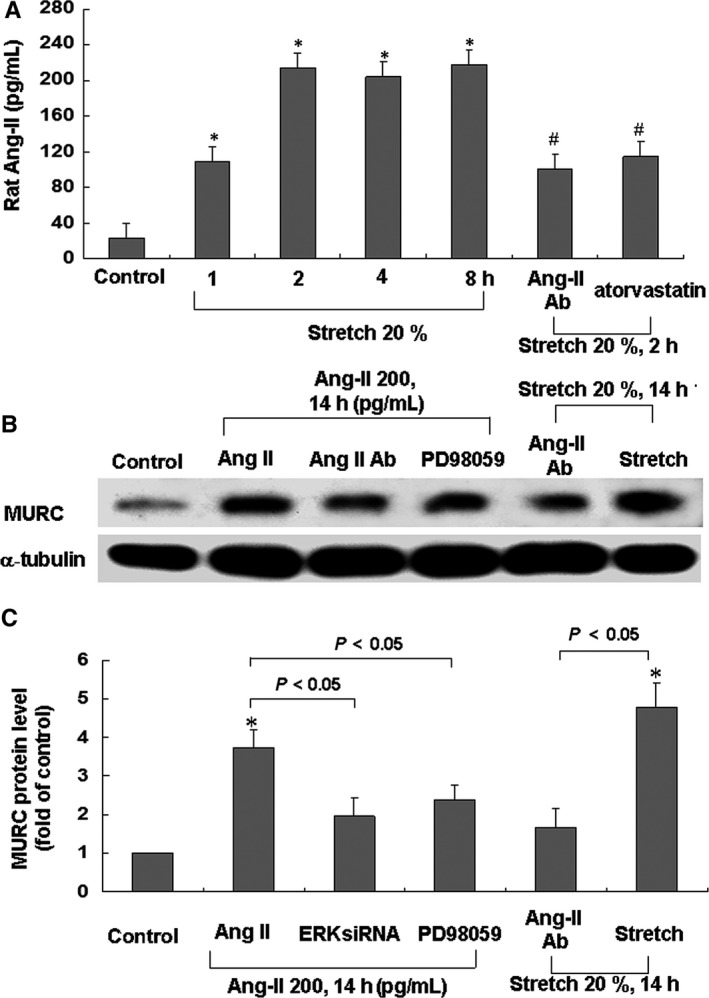
Effects of rat Ang II on MURC in cardiomyocytes. A, Release of rat Ang‐II from cardiomyocytes subjected to stretch for various periods of time (n = 3 per group). *P < 0.05 vs control. B, Representative Western blots for MURC in cardiomyocytes after exogenous administration of Ang II or subjected to stretch in the absence or presence of ERK inhibitor or Ang II antibody. C, Quantitative analysis of MURC protein levels. Values of MURC protein were normalized to match the *α*‐tubulin measurement and then expressed as a ratio of normalized values to the control cells (n = 3 per group). *P < 0.05 vs control

Two hours of cyclic stretch resulted in markedly enhanced the Ang II secretion from cardiomyocytes, and this effect remained after 8 h (Figure 5A). Adding the Ang II antibody or atorvastatin 30 minutes before stretch markedly reversed the expression of Ang II induced by stretch. These findings demonstrated that stretch induced secretion of Ang II from cardiomyocytes. To determine the direct effect of Ang II on cardiomyocyte MURC expression, Ang II at different concentrations was administrated to the culture medium for 14 hours. The effect of Ang II on the expression of MURC protein was dose‐dependent (Figure S4). As detailed in Figure 5B,C, adding the Ang II antibody or PD98059 reversed the MURC protein expression induced by exogenous administration of Ang II. Moreover, adding the Ang II monoclonal antibody 30 minutes prior to stretch markedly reduced the expression of MURC induced by stretch. These results indicated that the cyclic stretch that increased cardiomyocyte MURC expression was mediated by Ang II.

### 3.7 Stretch‐induced hypertrophy occurred through MURC

**Figure 6:**
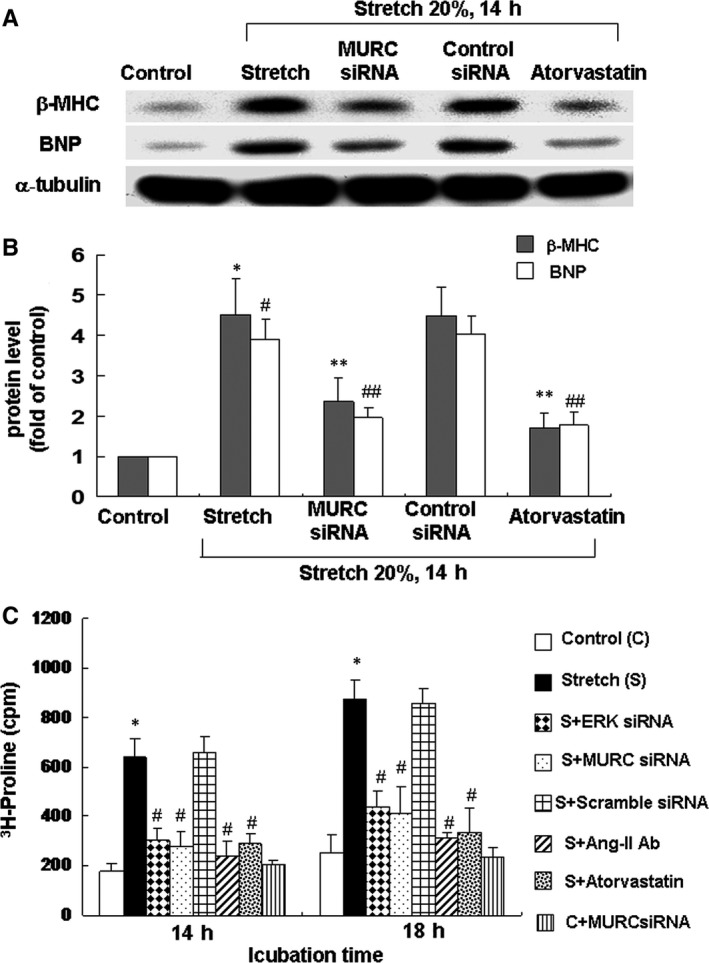
Effect of MURC on stretch‐induced hypertrophy in cardiomyocytes. A, Representative Western blots for β*‐*MHC and BNP in cardiomyocytes subject to stretch for 14 hours, with addition of siRNA before stretch. B, Quantitative analysis of β*‐*MHC and BNP protein levels. Values of β*‐*MHC and BNP protein were normalized to match the α‐tubulin measurement and then expressed as a ratio of normalized values to control cells (n = 3 per group). *P < 0.05 vs β‐MHC control. **P < 0.05 vs β*‐*MHC stretch. ^#^ *P < *0.05 vs BNP control. ^##^ *P < *0.05 vs BNP stretch. C, Incorporation of [^3^H]proline into cardiomyocytes increased after stretch for 14‐18 hours and addition of ERK or MURC siRNA, Ang II antibody and atorvastatin before stretch (n = 3 per group). *P < 0.05 vs control; ^#^ *P < *0.05 vs stretch

MURC siRNA was used to determine the role of MURC in hypertrophy under cyclic stretch. The stretch‐induced enhancement of MURC protein was significantly reduced after the treatment with MURC siRNA 24 hours before stretch (Figure S5). As shown in Figure 6A, pre‐treatment with MURC siRNA prior to stretch inhibited the stretch‐induced protein expression of hypertrophy markers β*‐*MHC and BNP. We discovered an increase in protein synthesis after stretch for 14‐18 hours, which represented a hypertrophic change in the cardiomyocytes (Figure 6C). Moreover, adding ERK or MURC siRNA, Ang II antibody or atorvastatin reversed the protein synthesis induced by stretch. We demonstrated that cardiomyocyte hypertrophy induced by stretch was mediated by MURC.

## 4 DISCUSSION

In this study, we obtained the following significant results. First, mechanical stress involving an in vivo AV shunt and in vitro cyclic stretch enhanced MURC expression in cardiomyocytes. Second, Ang II was involved in the MURC protein expression induced by stretch. Third, MURC protein expression was induced by cyclic stretch through the ERK MAPK pathway and SRF. Fourth, the cardiomyocyte hypertrophy induced by cyclic stretch was MURC dependent. Finally, atorvastatin inhibited MURC protein expression and cardiac hypertrophy under AV shunt and stretch conditions.

Our study demonstrated that cyclic stretch enhancement of Ang II and MURC expression was mediated by Ang II in cardiomyocytes. Feng et al reported that mechanical stretch increased the formation of Ang II in neonatal rat ventricular myocytes.^14^ Miyagawa et  al revealed that MURC was involved in the Ang II‐induced activity of matrix metalloproteinase‐9.^15^ In addition, we demonstrated in this study that stretch‐enhanced hypertrophy was reversed by Ang II antibody. Previous studies have demonstrated that Ang II could induce cardiomyocytes hypertrophy.^16^, ^17^ These results are consistent with the findings of this study.

We demonstrated that MURC was induced by cyclic stretch through the ERK MAPK pathway in cardiomyocytes. PD98059, the specific inhibitor of ERK, reversed the MURC protein expression induced by stretch. However, the specific inhibitors of JNK and p38 did not affect the stretch‐induced MURC protein expression. Naito et al noted that MURC mutant lacking coiled‐coil domain enhanced ERK activation.^7^ In another study, MURC modulated cardiomyocyte hypertrophy occurred through ERK activation.^11^ Tagawa et  al reported that overexpression of MURC led to the activation of ERK in C2C12 myoblasts.^18^ In addition, one study indicated that MURC knock out mice had enhanced the tumour necrosis factor‐α‐induced JNK activity.^15^ These results imply that MURC is closely linked with MAPK.

We demonstrated that MURC was induced by stretch in cardiomyocytes through SRF. Previously, we demonstrated that SRF was involved in the regulation of MURC under hypoxia.^13^ SRF is a transcription factor modulating the expression of the foetal cardiac gene. SRF plays a crucial role in the development of hypertrophy.^19^ A previous study revealed that MURC regulated the SRF‐mediated hypertrophy through the Ras homolog gene family, member A/Rho‐associated protein kinase pathway.^18^ Rangrez et  al reported that SRF led to cardiomyocyte hypertrophy through overexpression of dysbindin.^20^ Our results suggested that SRF is involved in MURC‐mediated hypertrophy under cyclic stretch in cardiomyocytes.

Our results also indicated that cardiomyocyte hypertrophy induced by stretch through Ang II, ERK and MURC. Li et  al demonstrated that WenxinKeli inhibited hypertrophy induced by Ang II in H9C2 cardiomyocytes.^21^ In addition, previous researchers have reported that myeloid differentiation 1 attenuated the Ang II‐induced hypertrophy in neonatal rat cardiomyocytes. Xiong et  al also showed that the MEK(mitogen‐activated protein kinase kinase)‐ERK 1/2 signalling pathway was involved in the antihypertrophic effect under Ang‐II stimuli.^22^ These results are consistent with our findings. MURC was demonstrated to play a vital role in cardiac hypertrophy.^11^ Schlegel et  al reported that elevated expression of G protein‐coupled receptor kinase 2 induced cardiac hypertrophy.^23^ In this study, we demonstrated that Ang II, ERK and MURC played a crucial role in hypertrophy under stretch conditions in cardiomyocytes. Atorvastatin was discovered to inhibit the cardiac hypertrophy induced by volume overload and cyclic stretch. Mechanical stress was previously demonstrated to enhance cardiac hypertrophy. Lee et  al showed that regulator of G protein signalling 2 inhibited the hypertrophy induced by pressure overload.^24^ In other research, Zhao et  al reported that the deletion of interleukin‐6 showed resistance to hypertrophy in response to pressure overload.^25^ Researchers in a previous study also reported that volume overload resulted in the cardiac hypertrophy.^26^ In addition, Jiang et  al demonstrated that Ang II type 1 receptor was involved in the cardiomyocyte hypertrophy under mechanical stretch conditions.^27^ These studies are consistent with our observations that cardiac hypertrophy was induced by mechanical stress. Moreover, in this study, atorvastatin reversed the hypertrophy induced by volume overload and stretch. Liang et  al indicated that atorvastatin attenuated cardiac hypertrophy under cold exposure.^28^ In another study, Wang et  al demonstrated that pressure overload‐induced right ventricular hypertrophy was reversed by the atorvastatin treatment.^29^ Moreover, one study also showed an inhibitory effect of atorvastatin on hypertrophy caused by parathyroid hormone 1‐34.^30^ These results together demonstrated the inhibitory effect of atorvastatin on cardiac hypertrophy.

In summary, our study indicates that mechanical stress enhances MURC expression in cardiomyocytes. MURC expression is induced by stretch through an Ang II, ERK and SRF pathway. Atorvastatin treatment attenuates the cardiac hypertrophy in response to volume overload and cyclic stretch. Our study indicates that atorvastatin can attenuate cardiomyocyte hypertrophy by inhibiting MURC induced by volume overload and stretch.

## CONFLICT OF INTERESTS STATEMENT

None.
