# Supplementary figures and images for: Effect of atorvastatin on cardiomyocyte hypertrophy through suppressing MURC induced by volume overload and cyclic stretch
Source: J Cell Mol Med. 2018 Dec 3;23(2):1406–14. doi: 10.1111/jcmm.14044 (PMC6349245; doi:10.1111/jcmm.14044)

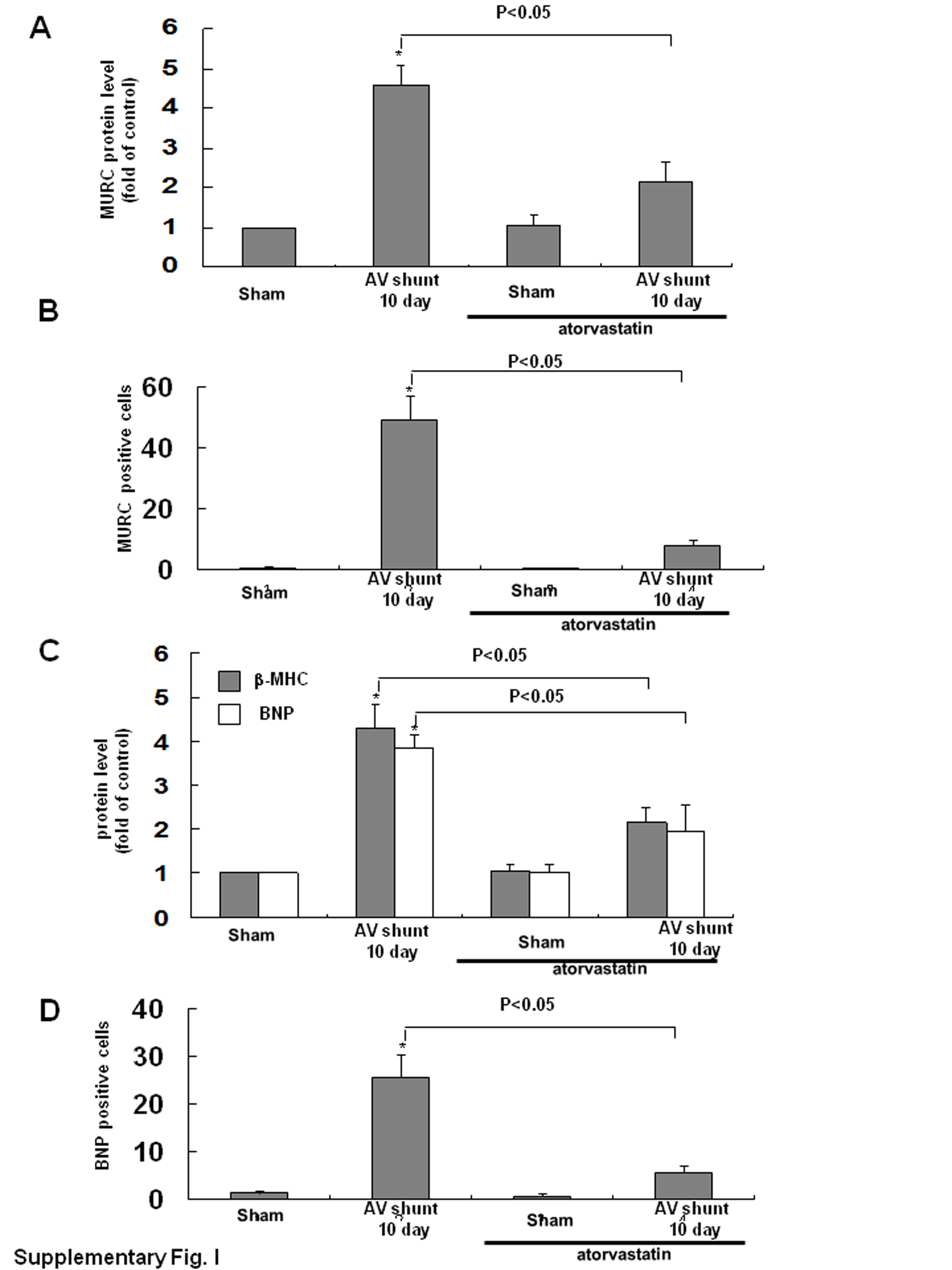

Supplement: Supplementary file 1 [file JCMM-23-1406-s001.tif]

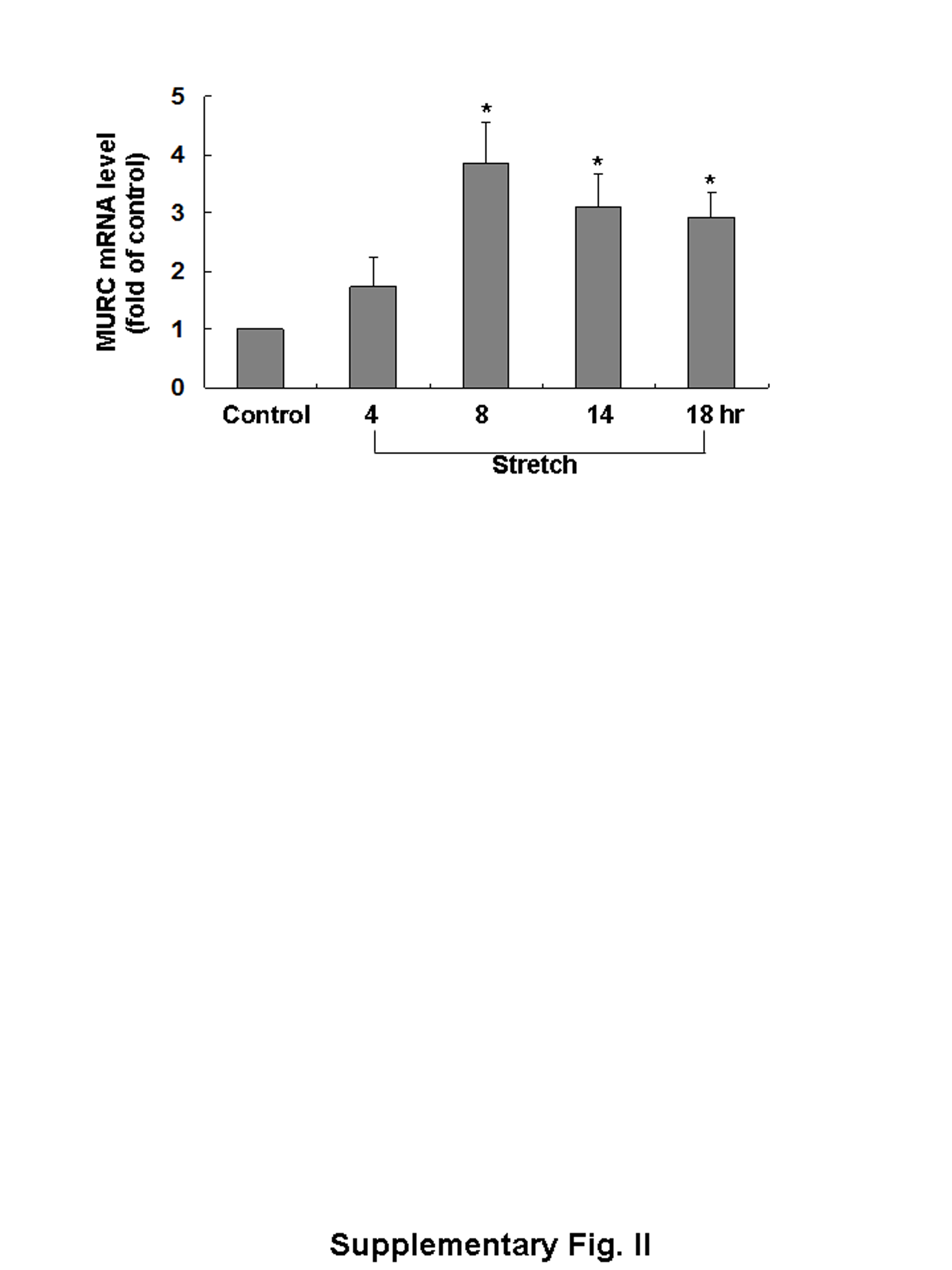

Supplement: Supplementary file 2 [file JCMM-23-1406-s002.tif]

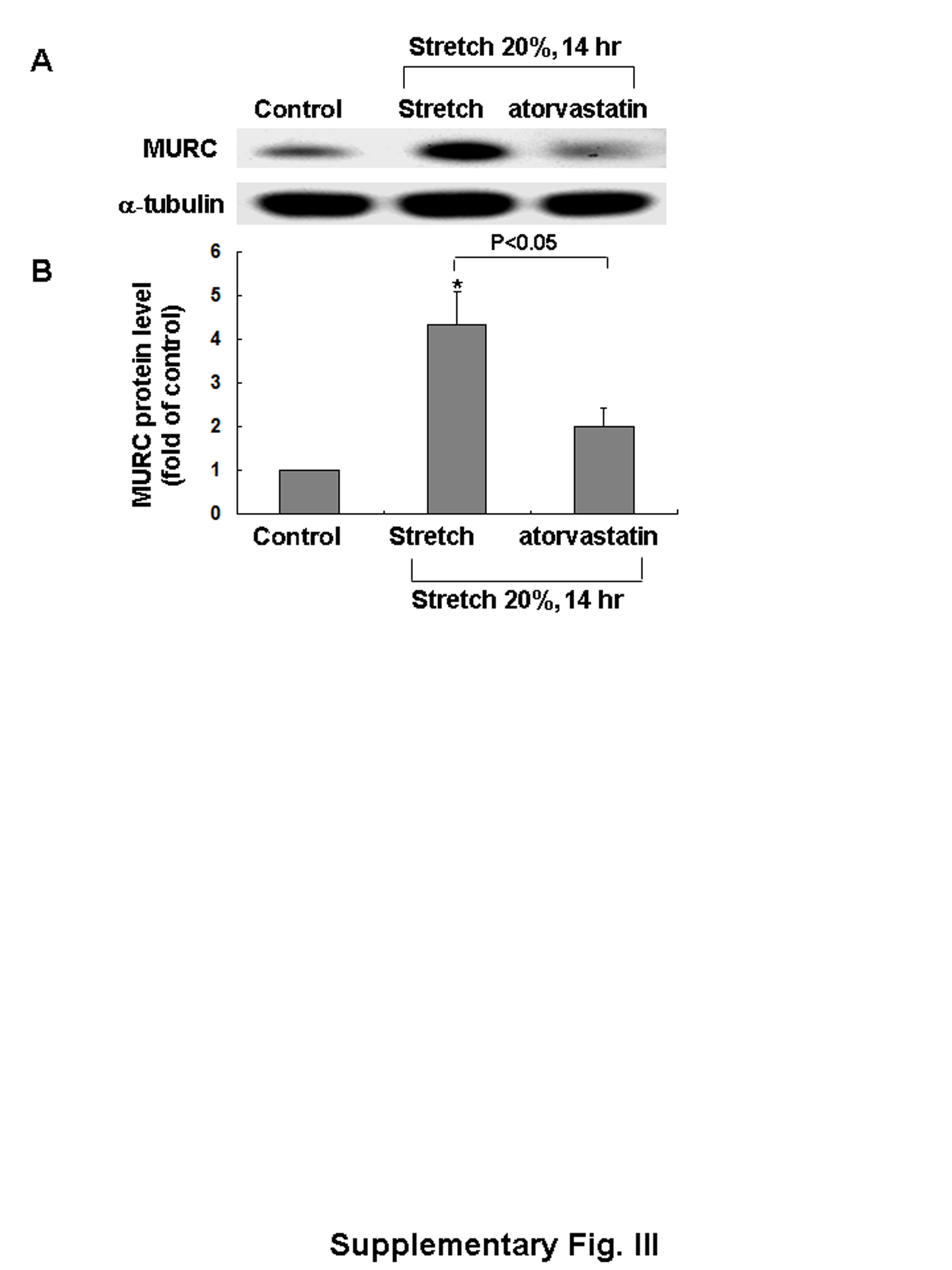

Supplement: Supplementary file 3 [file JCMM-23-1406-s003.tif]

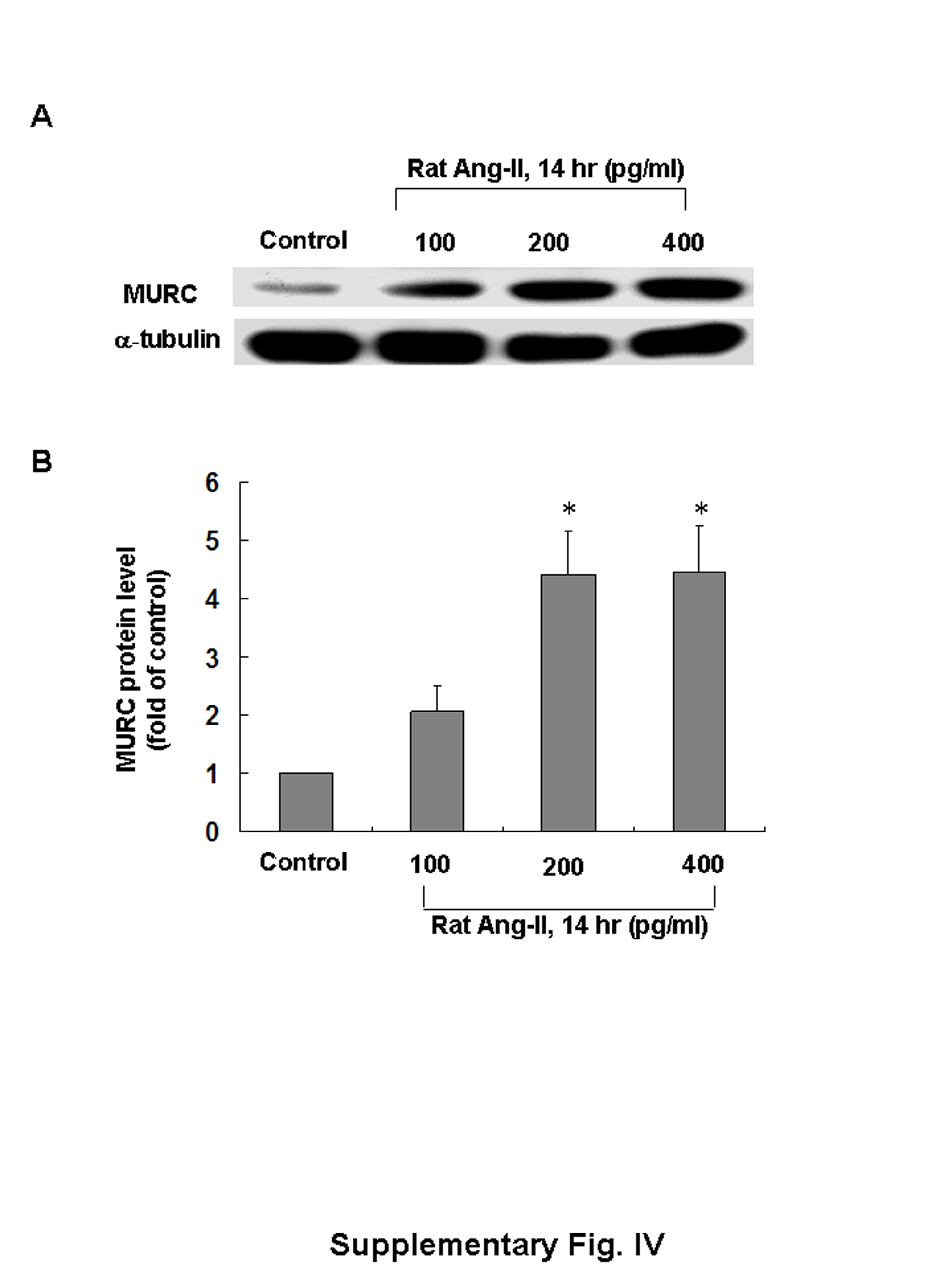

Supplement: Supplementary file 4 [file JCMM-23-1406-s004.tif]

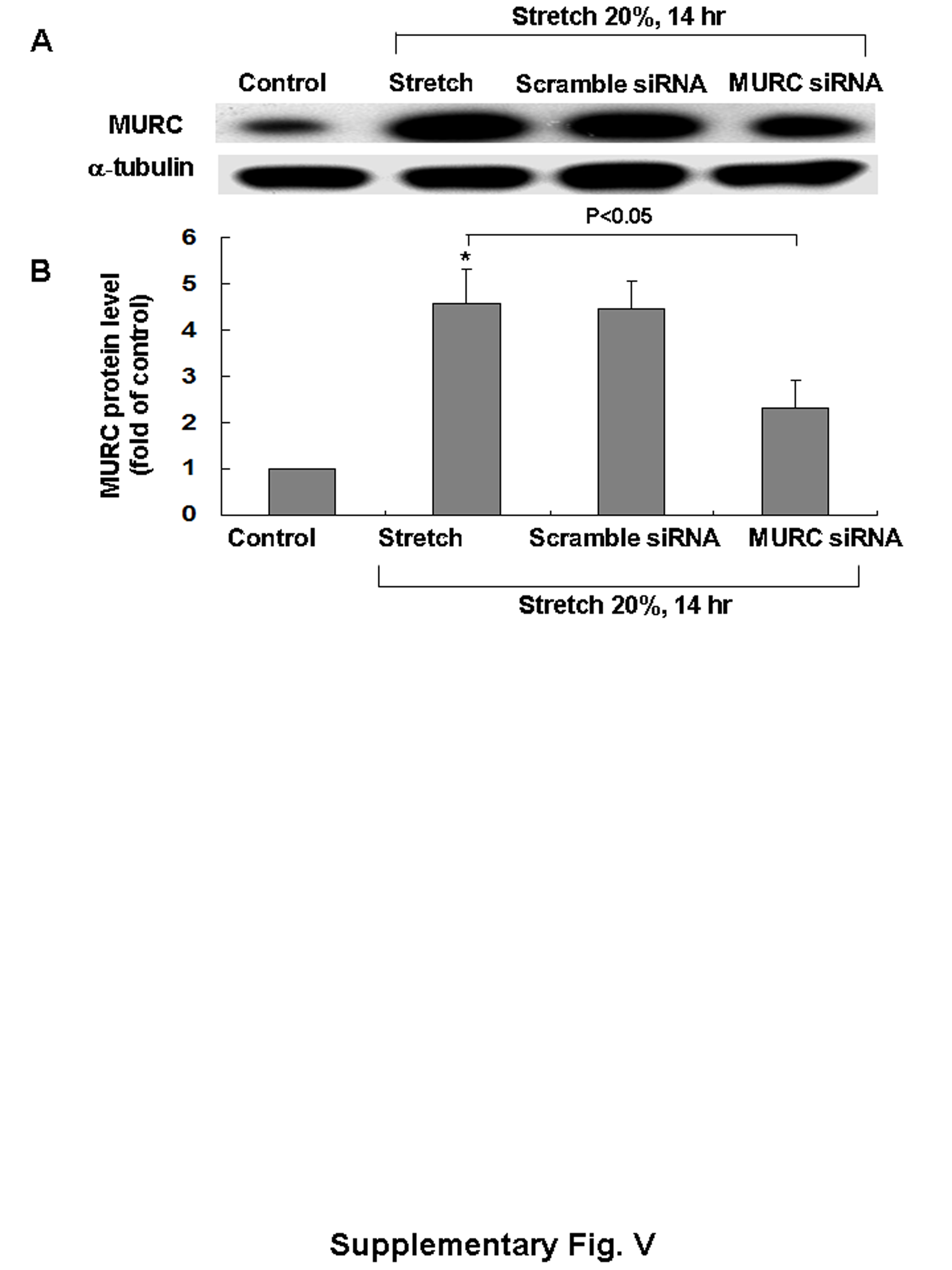

Supplement: Supplementary file 5 [file JCMM-23-1406-s005.tif]
