# Supplementary material for: Effect of atorvastatin on cardiomyocyte hypertrophy through suppressing MURC induced by volume overload and cyclic stretch
Source: J Cell Mol Med. 2018 Dec 3;23(2):1406–14. doi: 10.1111/jcmm.14044 (PMC6349245; doi:10.1111/jcmm.14044)
Supplement: Supplementary file 6 [file JCMM-23-1406-s006.doc]

**Supplementary Figure legends**

**SUPPLEMENTARY FIGURE I** Effect of atorvastatin on myocardial MURC, β−MHC and BNP protein expression after AV shunt for 10 days. (A) Quantitative analysis of MURC protein levels. Values for myocardia after induction of AV-shunt with or without treatment with atorvastatin. **P* < 0.05 *vs.* sham group. (n = 3 per group). (B) Quantitative analysis of MURC positive cells. **P* < 0.05 *vs.* sham group. (n = 3 per group). (C) Quantitative analysis of β−MHC and BNP protein levels. Values for myocardia after induction of AV-shunt with or without treatment with atorvastatin. **P* < 0.05 *vs.* sham group. (n = 3 per group). (D) Quantitative analysis of BNP positive cells. **P* < 0.05 *vs.* sham group. (n = 3 per group).

**SUPPLEMENTARY FIGURE II** Effect of cyclic stretch on MURC mRNA expression in cardiomyocytes. Quantitative analysis of MURC mRNA levels. Values for cardiomyocytes after stretch were normalized to match the GAPDH (glyceraldehyde-3-phosphate dehydrogenase) measurement and then expressed as a ratio of normalized values to mRNA in the control group (n = 3 per group). **P* < 0.05 *vs.* control.

**SUPPLEMENTARY FIGURE III** Effect of atorvastatin on MURC protein expression after cyclic stretch in cardiomyocytes. (A) Representative Western Blots for MURC protein levels in cardiomyocytes subjected to stretch in the absence or presence of atorvastatin. (B) Quantitative analysis of MURC protein levels. Values for cardiomyocytes after stretch were normalized to match the α-tubulin measurement and then expressed as a ratio of normalized values to protein in the control group (n = 3 per group). **P* < 0.05 *vs.* control.

**SUPPLEMENTARY FIGURE IV** Effect of Ang-II on MURC protein expression in cardiomyocytes. (A) Representative Western blots for MURC in cardiomyocytes after exogenous administration of Ang-II. (B) Quantitative analysis of MURC protein levels. Values for cardiomyocytes were normalized to match the *α*-tubulin measurement and then expressed as a ratio of normalized values to control cells (n = 3 per group). **P* < 0.05 *vs.* control.

**SUPPLEMENTARY FIGURE V** Effect of MURC siRNA on MURC protein expression in cardiomyocytes after cyclic stretch. (A) Representative Western Blots for MURC protein levels in cardiomyocytes subjected to stretch in the absence or presence of MURC siRNA. (B) Quantitative analysis of MURC protein levels. Values for cardiomyocytes after stretch were normalized to match the α-tubulin measurement and then expressed as a ratio of normalized values to protein in the control group (n = 3 per group). **P* < 0.05 *vs.* control.
